# Generalized Linear Quadratic Control for a Full Tracking Problem in Aviation

**DOI:** 10.3390/s20102955

**Published:** 2020-05-22

**Authors:** Franciszek Dul, Piotr Lichota, Artur Rusowicz

**Affiliations:** 1 Institute of Aeronautics and Applied Mechanics, Warsaw University of Technology, 00-665 Warsaw, Poland; piotr.lichota@pw.edu.pl; 2 Institute of Heat Engineering, Warsaw University of Technology, 00-665 Warsaw, Poland; artur.rusowicz@itc.pw.edu.pl

**Keywords:** control, LQR, system identification, trajectory tracking, aviation

## Abstract

In this paper, the full tracking problem in aircraft system identification and control is presented. Time domain output error method with maximum likelihood principle was used to perform system identification. The linear quadratic regulator (LQR)-based approach has been used for solving aviation full tracking problems in aviation. It has been shown that the generalized nonlinear LQR control is able to handle such problems even in case of inaccurate measurements and in the presence of moderate disturbances provided that the model of an aircraft is properly identified.

## 1. Introduction

The tracking problem is of great importance in advanced aircraft control [1,2,3,4,5,6,7]. It consists of determining the control for an aircraft that keeps its position close to the prescribed trajectory in some sense. The full tracking problem consists of keeping all the states of an aircraft (linear and angular velocities, position and orientation) close to the prescribed states for some time interval. The full tracking assures prescribed timing of an aircraft. There are two specific areas in aviation where tracking problems are of special interest:

- off-line identification from flight test data and dynamical model tuning e.g., for C and D class full flight simulators certification
- performing a flight along a prescribed path with appropriate timing using automatic control in real time.

Both these problems can be tackled by using a classical control systems methodology based on PID controllers [8,9,10,11,12,13]. However, developing a multidimensional PID controller is difficult because there is no theoretical background for them. Thus, such synthesis is somewhat intuitive, depends on rules of thumb and requires great experience from control system engineers. Another possibility is to use back-stepping or sliding mode control. In [14], a terminal sliding mode and back-stepping control was successfully implemented in a real-time unmanned aerial vehicle. In [15], a robust controller, based on linear feedback representation to reduce dynamic uncertainties and external disturbances was designed and implemented in real-time underactuaded system.

Modern control techniques, especially the optimal control theory, give a possibility of developing multidimensional controllers that are efficient and robust [16,17,18,19,20]. They are well suited for handling the tracking problems of very general types. In [21], instantaneous optimal control is used for robot trajectory tracking with input saturation. Three-D underactuated crates tracking with symplectic pseudospectral optimal control is presented in [22]. In [23], it was shown that an optimal periodic controller can be used for spacecraft formation keeping. Among those problems, the most difficult is the full tracking problem in which all states of an object have to be fitted. There are threemain types of control based on optimal control theory: linear quadratic control (LQR), linear quadratic Gauss control (LQG), and H-type controls (H_1_, H_2_ and H_∞_) [24,25,26,27]. The LQR and LQG controls are defined and realized in the time domain, whereas the H- techniques, being a generalization of classical loop shaping technique, are defined in the frequency domain [27]. For the tracking problem analyzed here, the time-domain approach seems to be more adequate, thus H-control will not be considered. Further, other types of control, e.g., nonlinear control (sliding control, describing function approach) will not be addressed here.

In this paper, an application of the generalized LQR control for nonlinear full tracking problems in aviation will be presented. It will be shown that such an extended version of LQR control can be used with success for real tracking problems even in the case of inaccurate measurements and with presence of uncertain physical disturbances, such as turbulence or unknown wind characteristics. The problem of fitting the full state of an aircraft to the airborne gathered flight data has been chosen for the illustration of the proposed methodology. It will be shown that if available measurements are sufficiently accurate, the generalized LQR control enables tracking the prescribed trajectory with sufficient accuracy for a long lasting flight, provided that model of an aircraft is adequate and accurate enough. The question of inaccurate measurements will also be considered with analysis of their impact on stability and efficiency of the LQR control. It will be also pointed out that the best results in using LQR control for aircraft trajectory tracking can be obtained if the aircraft model is developed through system identification [28,29].

This distinguishes our study from other aeronautical full tracking problems, as it incorporates system identification models obtained for large aircraft C- and D-class full flight simulators in the LQR design process. However, gathering the data from a flight test campaign is time consuming and thus very costly. For the certified full flight simulators, this is not a serious limitation as no other means (e.g., computational fluid dynamics) can be used to obtain dynamical models as stated in the EASA or FAA regulations [30,31].

**Figure 1:**
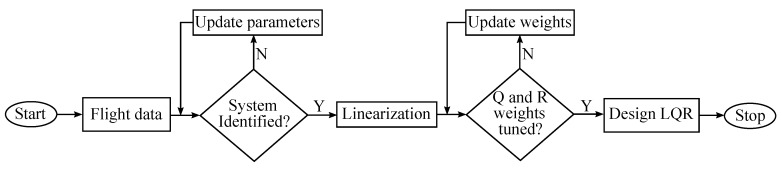
LQR design flowchart.

The paper is organized as follows. In Section 2, the general tracking problem is presented with application to the airplane model identified from flight data. Section 3 describes a methodology of using the LQR control in the full tracking problems. In Section 4, an example of a full tracking problem for generic cargo aircraft with an initially identified model will be formulated. Results are presented in Section 5. Efficiency of LQR control in aircraft tracking problems and the impact of inaccurate measurements on its effectiveness and stability is shown in that part. In Section 6 we end the paper with a short summary of conclusions. The overall LQR design process is presented in Figure 1.

## 2. Problem Formulation

Development, construction and putting into operation a modern flight simulators is a complex process that requires, among others, developing an accurate and reliable physical and mathematical model of an aircraft and, according to the regulations, a verification of quality of the model based on real data gathered in flight.

### 2.1. System Identification

Performing a flight test campaign in order to design a computational method or validate the initial results would be very costly. Thus, a common approach is to build a simulation model of the aircraft (with uncertainties, noise effects, etc.) and to analyze its response [32,33,34]. This method was used in this study and the Basic AirCraft Model (BACM) developed at the DLR German Aerospace Center was representing a real aircraft. The aircraft was excited, its response was registered and on the basis of this data, aerodynamic coefficients of the object were estimated.

**Figure 2:**
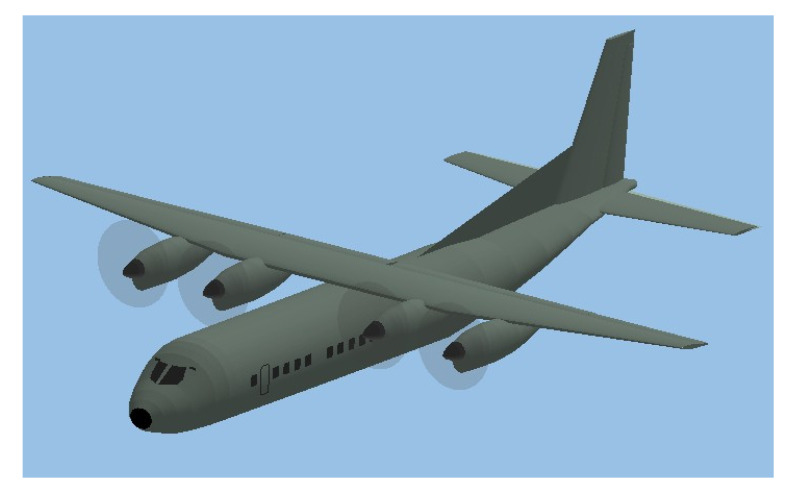
Aircraft model.

The aircraft is presented in Figure 2, whilst its technical and performance data is shown in Table 1.

The system was identified using ASIA (Advanced System Identification Application), a Matlab toolbox developed at WUT. The software uses the time-domain output error domain method in order to find unknown parameters of the system under study, i.e., it minimizes errors between the measurements z and the estimated model $\hat{y}$. In order to perform this task, a maximum likelihood principle is used which means that the conditional probability *p* of observing measurements for a selected set of system parameters Θ is maximized:

(1) $$\hat{\Theta }=\arg\left\{\max_{\Theta }p\left(z|\Theta \right)\right\}$$

When multivariate normal distribution is selected and measurement errors are uncorrelated, it can be shown that the maximization task can be replaced by minimization of the following cost function *J*:

(2) $$J\left(\Theta \right)=\prod_{i=1}^{n}\left(\frac{1}{N}\sum_{k=1}^{N}{\left(z_{i}\left(t_{k}\right)-y_{i}\left(t_{k}\right)\right)}^{2}\right)$$

where k=1,...,N denotes time point and i=1,...,n the measured flight parameter.

In the performed task, the outputs were linear *u*, *v*, *w* and angular velocity components *p*, *q*, *r*, roll and pitch angles ϕ, θ and linear accelerations $a_{x}$, $a_{y}$, $a_{z}$, which is a typical set for flight dynamics problems [35]. A two point-model was used for describing an estimated object:

(3) $$\begin{array}{c} C_{L}=C_{L_{0}}+C_{L_{WB_{\alpha }}}\alpha +\frac{S_{H}}{S}\left(C_{L_{H_{\alpha H}}}\alpha_{H}+C_{L_{H_{\delta H}}}\delta_{H}\right)\\ C_{D}=C_{D_{0}}+kC_{L}^{2}\\ C_{m}=C_{m_{0}}-\frac{x_{LH}S_{H}}{cS}\left(C_{L_{H_{\alpha H}}}\alpha_{H}+C_{L_{H_{\delta H}}}\delta_{H}\right)+C_{m_{q}}q\\ C_{Y}=C_{Y_{0}}+C_{Y_{\beta }}\beta +\left(C_{Y_{p}}+C_{Y_{p\alpha }}\alpha \right)+\left(C_{Y_{r}}+C_{Yr_{\alpha }}\alpha \right)r+C_{Y_{\delta A}}\delta_{A}+C_{Y_{\delta R}}\delta_{R}\\ C_{l}=C_{l_{0}}+C_{l_{\beta }}\beta +\left(C_{l_{p}}+C_{l_{p\alpha }}\alpha \right)+\left(C_{l_{r}}+C_{lr_{\alpha }}\alpha \right)r+C_{l_{\delta A}}\delta_{A}+C_{l_{\delta R}}\delta_{R}\\ C_{n}=C_{n_{0}}+C_{n_{\beta }}\beta +\left(C_{n_{p}}+C_{n_{p\alpha }}\alpha \right)+\left(C_{n_{r}}+C_{nr_{\alpha }}\alpha \right)r+C_{n_{\delta A}}\delta_{A}+C_{n_{\delta R}}\delta_{R} \end{array}$$

where $C_{\left(\right)}$ denotes aerodynamic coefficient, α and β are angle of attack and sideslip angle, *S* is the wing area, *c* mean aerodynamic chord whereas WB and *H* indices denote wing-body components and horizontal tail. Aerodynamic derivatives can be obtained e.g., by using CFD methods. However, these methods are numerically expensive [36], therefore, in this work, initial values of the aerodynamic derivatives were obtained from previous studies [37].

During the identification maneuver each flight surface was able to switch between three states (zero value, negative amplitude and positive amplitude). The inputs were applied separately to each control; elevator was excited with a 3-2-1-1 signal, ailerons with 1-2-1 control and rudder with a doublet. For the elevator, the switching time was 1 s, whereas for aileron and rudder it was 2 s. Inputs amplitudes were 2${}^{\circ }$, 8${}^{\circ }$ and 6${}^{\circ }$ respectively. Each excitation was preceded with an unperturbed trimmed flight lasting 5 s. The switching times were selected accordingly to Marchand method [29] in order to provide a sufficient amount of information stored in the output signals. Trimmed flight at the beginning of the manoeuvre allowed to estimate static components. The experiment was performed in good meteorological conditions in order to eliminate process noise due to turbulence. The experiment was performed multiple times at the same trim point in order to reduce random errors.

To identify the system, first, the flight parameters associated with the longitudinal motion (*u*, α, *q*, θ, $a_{x}$, $a_{z}$) were used for parameter estimation and those associated with lateral-directional were kept fixed (β, *p*, *r*, ϕ, $a_{y}$). Then, lateral-directional flight variables were used and longitudinal motion parameters were kept fixed. After obtaining parameter estimates from both cases, all output signals were used to obtain final estimates. It was possible to use this approach sice for aircraft, there usually is no strong coupling between longitudinal and lateral motion. This allowed to limit the identification time and to raise its accuracy, as the model structure was not overdetermined. As can be observed, performing system identification task required understating of the object dynamics and cannot be automatized in a general case.

**Figure 3:**
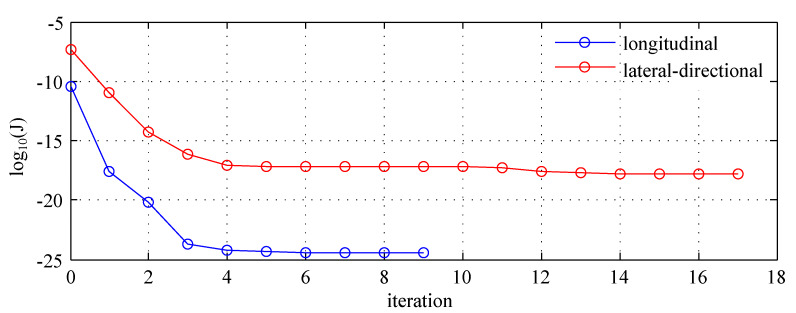
Cost function.

Relative change of the cost function smaller than 0.001 was the stop criterion for all cases. The cost function history when using longitudinal and lateral-directional flight parameters is presented in Figure 3, where 0 denotes initial iteration. When estimating the full model combined data from longitudinal and lateral-directional decreased initial cost function value to $J=3.57\times 10^{-34}$ and the final solution was obtained after one iteration, leading to $J=3.56\times 10^{-34}$.

Relative standard deviations for all identified aerodynamic coefficients were below 10%, as can be seen in Table 2 (OEM). The results obtained by using Nonlinear Linear Squares (NLS) are also presented in the Table. It can be observed that the system was identified with much higher accuracy when output error method was used. This can be especially seen when comparing second order cross-coupling derivatives. For model structure determination, a backward elimination method was used.

The identified aircraft was found to be statically and dynamically stable. Its longitudinal motion was described by two pairs of complex conjugates corresponding to short-period and phugoid modes. In the lateral-directional motion a complex conjugate pair described dutch roll motion, whilst two real numbers were related to roll subsidence and spiral modes.

The data for system identification was sampled at 50 Hz as the upper limit for aircraft dynamic modes is usually below 2 Hz and anti-aliasing filters are used (with the same cut-off frequency). The same sampling was used in the rest of the study.

### 2.2. Full Tracking Problem

In the course of aircraft model verification, the full tracking problem of control arises in a natural manner. It can be formulated generally as follows:

Given the state and controls of an aircraft as well as environment conditions gathered in flight, we find the controls that assures the state computed by the aircraft’s model to be close to the recorded state in the sense appropriately defined. Optionally, controls that assures fitting of state may be required to be close to those of an aircraft gathered in flight.

The success in handling the full tracking problem depends essentially on available data and their accuracy. It is necessary that all states of an aircraft are available: linear and angular velocities, position and attitude; other states might also be considered, e.g., propulsion system states, etc. The control system based on LQR should then compute controls $u^{*}$ that minimize the local error of the state with respect to the recorded state $x_{r}$

(4) $$||x\left(t\right)-x_{r}\left(t\right)\left|\right|\to \min$$

Measurements of the whole state vector of an aircraft are rarely available in practice, because angular rates and side velocity are not of interest for flight control and pilots. Moreover, only the geographic positions (latitude and longitude) are known, not the cartesian coordinates in WGS reference systems. However, in system identification and tuning based on flight tests a special equipment is used for measuring remaining state variables: gyroscopic platforms for measuring angular velocities and vanes for measuring angle of attack (AOA) and sideslip angle that are equivalent with the vertical and side velocities. Environmental state is usually not complete: the wind velocity and direction can be taken from GPS, however, the vertical air velocity is usually not known. Stochastic characteristics of turbulence are also unknown; they can be estimated qualitatively based on assumed models of turbulence (usually Dryden or von Karman models). Air density ρ, which is of primary importance for accurate aircraft modeling, can be estimated only indirectly from gas equation based on air temperature *t* and static air pressure *p* that are measured routinely

(5) ρ=p/(R(t+273.5))

where *R* = 287.058 is the specific gas constant.

Since the tracking problem is an evolution processes and recorded data have the form of long sampled time series, the time-domain methods are preferred. Moreover, the dimension of problem, being routinely greater than twelve, makes the use of standard methods based on PID regulators very problematic. This implies that the use of LQR optimal control seems to be the best choice for solving the full tracking problem considered here.

## 3. LQR Approach

Full tracking is a problem that can be handled by LQR optimal control. The LQR control consists in synthesizing the feedback control [38]:

(6) u=−Kx

where *x* and *u* are state and control vectors, and *K* is the gain matrix.

The aim is to find an optimal control $u^{*}$ by solving the constrained optimization problem

(7) $$u^{*}=\arg\left\{\min_{u}J\left(x,u\right)\right\}$$

where J(x,u) is the quadratic performance index

(8) $$J=\int_{0}^{\infty }\left(x^{T}Qx+u^{T}Ru\right)dt$$

with *Q* > 0 and *R* > 0 weighting matrices for state and control, respectively. Their constrains are given by the linear plant model

(9) $$\frac{dx}{dt}=Ax+Bu$$

where *A* and *B* are the state and control matrices.

In LQR control, the plant has to be linear, no disturbances are allowed and time horizon of control is large. Due to those assumptions, a solution in closed form can be obtained by solving the stationary algebraic Riccati matrix equation:

(10) $$A^{T}P+PA-PBR^{-1}B^{T}P+Q=0$$

and stabilizing solution *P* > 0 is used to synthesize the gain matrix *K*

(11) $$K=R^{-1}B^{T}P$$

The LQR controller has a large gain margin and 60${}^{\circ }$ phase margin that make it very attractive, from both: theoretical and practical points of view [38]. Moreover, the LQR control can also be generalized to nonlinear problems with a nonlinear plant model, provided that the state function vector field is linearizable, i.e., its jacobians withe respect to state *x* and control *u* exist.

The main disadvantage of the LQR is the necessity of measuring the full state *x* of the plant for determining the feedback. It also does not take into account disturbances, neither physical (atmospheric, measurement) nor modeling ones. Moreover, there are no established methods for selecting the weighting matrices *Q* and *R*, whereas success in using the LQR controller depends strongly on choosing and tuning them.

In the considered full tracking problem, all state components are available. Very good robustness properties of LQR give a chance that physical and measurement disturbances will be handled efficiently if they are not too large. Further, the modeling errors may be not very important if the aircraft model is adequate and identified with high accuracy. Thus, the main question concerning the use of LQR for the full tracking problem is whether the weighting matrices *Q* and *R* that assure stable control $u^{*}$ of the given nonlinear system can always be found, and how to select them.

Keeping in mind the above conclusions, the use of an extended LQR control seems to be useful for handling the general tracking problem, where all the states are available.

## 4. Full Tracking Problems

The problem is to establish the control for a cargo aircraft to fly close to the prescribed trajectory with requirement of time synchronization, that is, aircraft should not only follow the geometry of the trajectory, but it should also be close to the trajectory in prescribed moments of time. Such a problem is thus the full tracking problem in the sense defined in Section 2. From a practical point of view, this is a task of precise flight realization that assures safe flight in airspace with high air traffic.

In the study, basic control and navigation equipment were available, including GPS data. Angular rates from a gyroscopic platform mounted at center of mass, angle of attack and sideslip angle from five hole differential pressure probes were given as well. The total thrust of four engines was registered.

Thus, the available measurements were: total velocity *V*, angle of attack α and sideslip angle β, vertical velocity $V_{w}$, angular velocities: *p*, *q*, *r*, position *X*, *Y*, *Z*, latitude φ and longitude λ, altitude (barometric or radio) *h*, attitude angles: pitch θ, roll ϕ and yaw (heading) ψ, total thrust *T*, and the corresponding measurement vector was:

(12) $$y={\left[\begin{array}{ccccccccccccc} V & \alpha & \beta & V_{w} & q & p & p & \lambda & \varphi & \phi & \theta & \psi & T \end{array}\right]}^{T}$$

To describe the problem nonlinear rigid body aircraft model was used [39]:

(13) $$\frac{dx}{dt}=f\left(x,u;\eta \right)$$

where *x* is the state vector:

(14) $$x={\left[\begin{array}{cccccccccccc} u & v & w & p & q & r & X & Y & Z & \phi & \theta & \psi \end{array}\right]}^{T}$$

*u* is the control vector:

(15) $$u={\left[\begin{array}{cccc} \delta_{E} & \delta_{A} & \delta_{R} & \delta_{T} \end{array}\right]}^{T}$$

and $\delta_{E}$, $\delta_{A}$, $\delta_{R}$ are the elevator, aileron, rudder deflections, whilst $\delta_{T}$ is the engine throttle position (assumed the same for all engines), and η is a given environment state vector:

(16) $$\eta ={\left[\begin{array}{ccc} V_{wind} & \Theta_{wind} & \epsilon_{turb} \end{array}\right]}^{T}$$

containing wind velocity $V_{wind}$, direction $\Theta_{wind}$ and turbulence components $\epsilon_{turb}$.

The LQR was used to find a control $u^{*}$ that for the model minimized the performance index:

(17) $$J=\int_{0}^{\infty }\left(e^{T}Qe+u^{T}Ru\right)dt$$

where *Q*>0 and *R*>0 are the weighting matrices and *e* is the trajectory error:

(18) $$e=x-x_{tr}$$

and $x_{tr}$ is the prescribed trajectory.

Equation (17) was solved analogously to Equation (8)—instead of states *x* the trajectory errors *e* were used. The prescribed trajectory $x_{tr}$ was obtained from the measurement vector *y* by transforming *V*, α, β and $V_{w}$ variables to the *u*, *v*, *w* velocity components and geographic coordinates latitude, longitude and altitude to the WGS coordinates *X*, *Y*, *Z*. Thrust was not included in trajectory.

**Figure 4:**
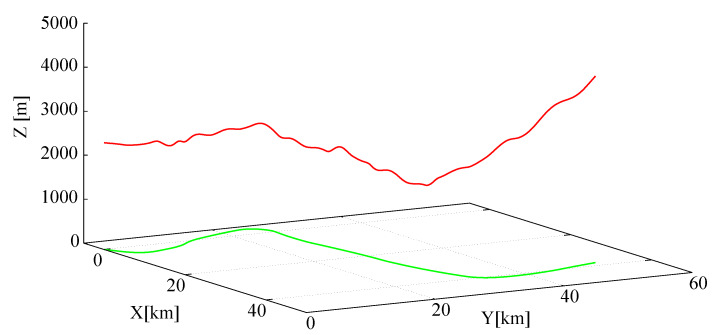
Exemplary trajectory.

The trajectory $x_{tr}$ was generated by running the model with assumed control $u_{pr}$ and undisturbed standard atmospheric conditions: $V_{wind}$ = 0, no turbulence. Sampled time series were recorded at 50 Hz for 600 s lasting flights. Trajectories were chosen according to the typical characteristics of the cargo aircraft flights, that is, they are smooth with only moderate turns, rolls, climbing and descending rates. No aerobatic maneuvers were allowed. In Figure 4, an exemplary trajectory (only its geometric part–flight path) is shown.

A linearized model of an aircraft for the extended LQR control was obtained by computing jacobians of model equations:

(19) $$\begin{array}{c} A\left(x\right)=\frac{\partial f(x,u)}{\partial x}\\ B\left(x\right)=\frac{\partial f(x,u)}{\partial u} \end{array}$$

in which matrices *A* and *B* depend on actual state *x*. Jacobians were computed numerically by fourth order finite difference approximation. The given model was linear about its parameters.

As said earlier, the key point in using LQR control is a selection of weighting matrices *Q* and *R*. They were assumed diagonal and constant during the flight. Since there are no general rules for choosing *Q* and *R*, an experience-based procedure was used for this purpose. A good selection of the weighting matrices *Q* and *R* is crucial, because LQR control is sensitive to them. Improper *Q* and *R* element values result either in poor tracking or instability of aircraft motion. This is especially true for nonlinear extension of LQR, in which matrices A and B are not constant.

Diagonal element values in *Q* and *R* were bounded from the above to assure control stability. Their very high values result in control oscillations that lead to unstable flight, whereas very small values of the *Q* and *R* elements result in poor tracking and quick aircraft departure from the prescribed trajectory. Designing *Q* and *R* is not obvious, because their values are related implicitly to one another in an unknown manner. Some experience-based procedure has to be used for choosing them.

In this study, a semi-automatic iterative procedure for finding the *Q* and *R* matrices was used. Initially, low values of diagonal elements of *Q* and *R* were set (100). Those values usually do not assure good tracking (low errors), but stability of aircraft motion is preserved. Then, several runs were made with increased (twice) values of individual elements Q(i,i). This led to a reduction of the tracking error for corresponding state x(i). Similarly, increasing of the R(j,j) allowed to establish high authority of controls without raising of instabilities. The process of increasing *Q* and *R* elements values was continued until instabilities of aircraft motion or controls deflections were detected. Then, selected elements in *Q* or *R* matrices were decreased by a few percent points (5%) until instabilities disappeared.

Using the above procedure, the following values of the *Q* and *R* matrices were designed for the considered case:

$$\begin{array}{c} Q=\mathrm{diag}\left(\begin{array}{cccccccccccc} 1\times 10^{8} & 0 & 0 & 1\times 10^{2} & 1\times 10^{2} & 1\times 10^{2} & 0 & 0 & 0 & 1\times 10^{9} & 1\times 10^{9} & 1\times 10^{9} \end{array}\right) \end{array}$$

$$\begin{array}{c} R=\mathrm{diag}\left(\begin{array}{cccc} 1\times 10^{5} & 1\times 10^{5} & 1\times 10^{5} & 1\times 10^{4} \end{array}\right) \end{array}$$

With this method, usually a few tens of runs are necessary for proper *Q* and *R* tuning. This tuning procedure, although laborious, enables one to design *Q* and *R* matrices that provide good tracking properties and stability of aircraft motion. Automatization of this process is considered in further work.

It is also possible to use a Matlab Simulink Design Optimization library to design *Q* and *R* matrices automatically. However, one of the additional assumptions in this project was not to use additional commercial libraries. Thus, neither Matlab Simulink Design Optimization nor Aerospace Blockset/Toolbox were used.

Due to inherent nonlinearity of tracking problems formulated above the effectiveness and robustness of the generalized LQR control can be judged only qualitatively using numerical simulations. LQR control robustness was assessed by introduction of disturbed atmospheric conditions, i.e., non-zero wind and turbulence intensity in the data.

To design LQR and perform the evaluations in-house-developed C++ code was used. No specialist or commercial libraries were necessary to perform this task. For data visualization, the results were exported to MATLAB.

## 5. Results

### 5.1. Ideal Measurement and Calm Atmosphere

**Figure 5:**
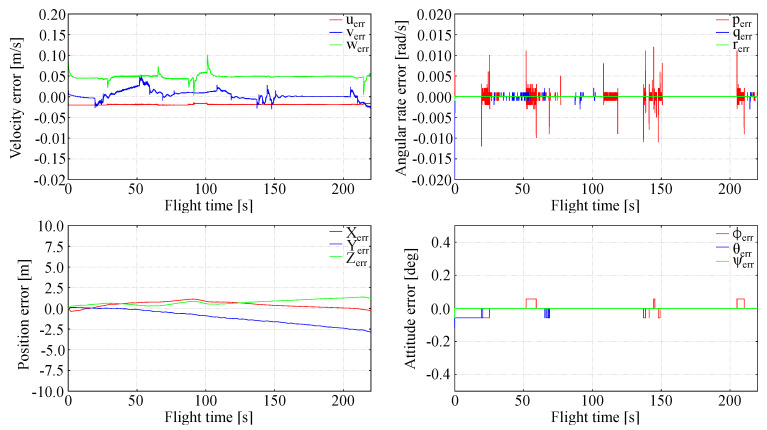
Full tracking errors, quiet atmosphere.

In order to assess the stability of the extended LQR control and tracking accuracy for long lasting flights, a case with no atmospheric disturbances was analyzed. For this purpose, the following errors were evaluated: the local errors e(t) and their maxima. In Figure 5 full state tracking error is shown.

**Figure 6:**
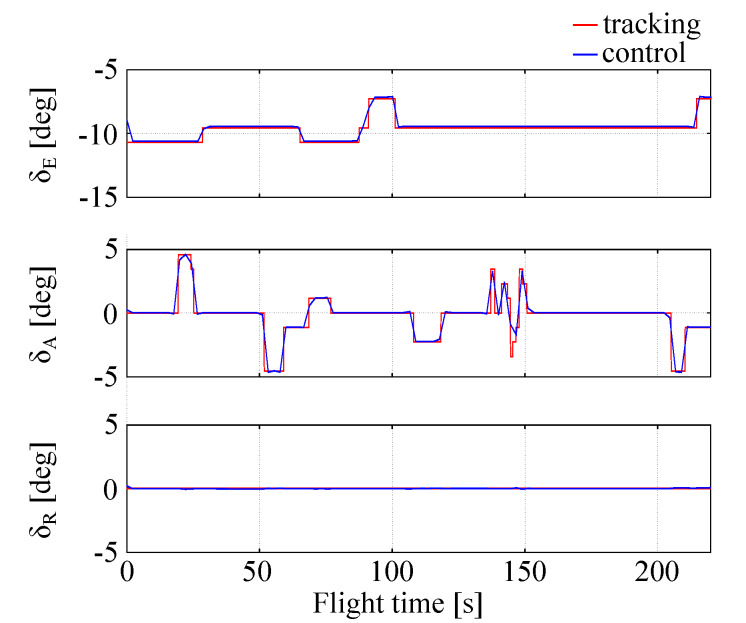
Controls errors.

It is evident that the full tracking using LQR is very effective in the considered case. The local errors e(t) are low (few percents of the state components nominal values). The position errors are of a few meters, which is achievable only if velocity errors are small. The flight is stable, there are no state variables oscillations. Attitude errors for roll, pitch and heading (yaw) are very small being on the order of one percent. The angular rates are very small. The LQR generated controls (elevator, aileron and rudder deflections) are in excellent agreement with those recorded in trajectory generating flights (Figure 6). This means that the generalized nonlinear LQR control seems to be an effective tool for the full tracking problems.

Results of these tests show that the generalized LQR control used for the full tracking problem is stable enough and assures accurate tracking of the prescribed trajectory in the absence of external disturbances, e.g., due to atmosphere.

### 5.2. Measurement and Atmospheric Disturbances

The aim of this test case was to check the robustness of the extended LQR control in the tracking problem. There are two disturbance types in the considered problem: measurement and physical disturbances. Modeling errors are assumed to be small due to the prior identification of the aircraft model.

When measurement and process noise are taken into account, the aircraft equation of motion can be presented as [29]:

(20) $$\begin{array}{c} \dot{x}=f\left(x,u\right)+v_{t}\\ y=g\left(x,u\right)+v_{m} \end{array}$$

where f(x,u) and g(x,u) are nonlinear real valued vector function, whereas $v_{m}$ and $v_{t}$ represent measurement and process noise (turbulence), respectively. In case of ideal measurements, $v_{m}$=0. Similarly, when process noise is not taken into account $v_{t}$=0.

The measurement disturbances concern both state and environment variables. Typical error values are presented in Table 3.

In order to assess the generalized LQR control robustness with respect to measurement disturbances, randomly generated disturbances with zero mean values and variances proportional to these from Table 3 were added to the measurement vector *y* as a realization of stationary stochastic process.

There were no essential differences in LQR control comparing to that described in testing stability and accuracy. Increment of the disturbances level resulted in roughly proportional distortion of tracking accuracy whereas stability of aircraft flight was no influenced. This means that LQR control used for the tracking problem was robust enough with respect to the measurement errors. The stability of LQR did not depend on measurement errors.

Generalized LQR control robustness assessment with respect to atmospheric disturbances concerned two cases: irregular disturbances with zero mean value represented by turbulence with assumed characteristics and intensities and systematic disturbances represented by constant wind or constant vertical wind flow.

**Figure 7:**
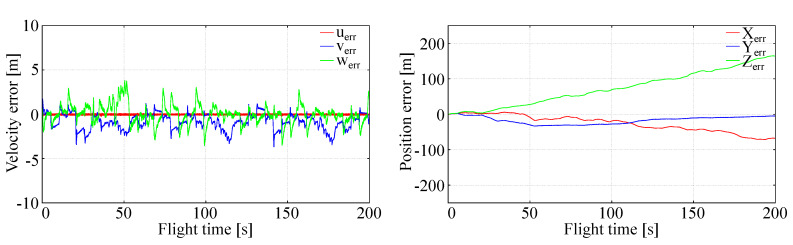
Full tracking in turbulent conditions.

In the case of irregular disturbances, the omnidirectional turbulence of Dryden type has been added with moderate intensity of 1–3 m/s. There were no essential differences in LQR control comparing to that described in testing stability and accuracy. Increasing the turbulence intensity resulted in a distortion of tracking accuracy consisting in oscillations of aircraft around trajectory, but as can be seen from Figure 7, departure of the aircraft from the trajectory was moderate and caused mainly by climbing due to nonzero mean of vertical velocity disturbance. Stability of the aircraft flight was not lost. This means that LQR control used for the tracking problem is robust enough with respect to homogeneous physical disturbances.

**Figure 8:**
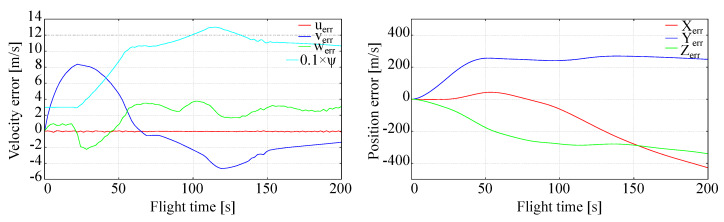
Full tracking in constant wind presence.

In the case of constant disturbances, the horizontal wind was added with typical velocity $V_{wind}$ = 50 km/h at any altitude, direction of which was constant, $\Theta_{wind}$ = 100${}^{\circ }$, so it was initially east relative to the aircraft and then turned to be north from t = 50 s flight time, becoming head due to the right aircraft turn performed as can be seen in Figure 8. Systematic error of side velocity caused by aircraft turn was observed. Side error position stabilized when wind became head, but longitudinal and vertical error positions started to increase as presented in Figure 8. Although the departure of an aircraft from the trajectory is significant, LQR controller tries to keep it as low as possible, and there are no instabilities of aircraft flight. This means that LQR control used for the tracking problem is robust enough even with respect to the systematic atmospheric disturbances.

**Figure 9:**
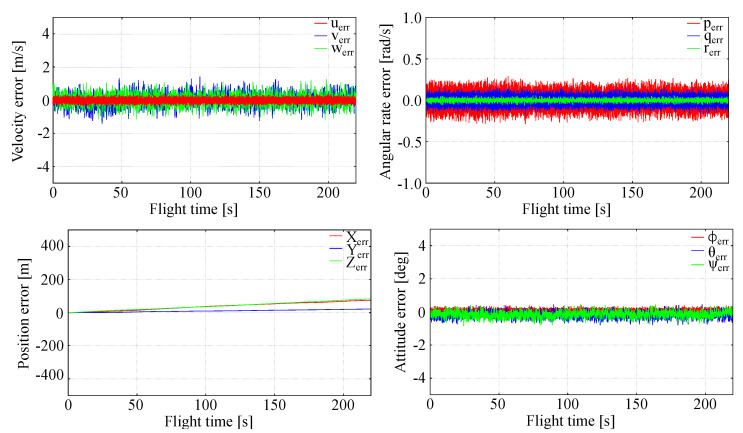
Full tracking errors—flight parameters disturbed by 1 unit (Case 1).

**Figure 10:**
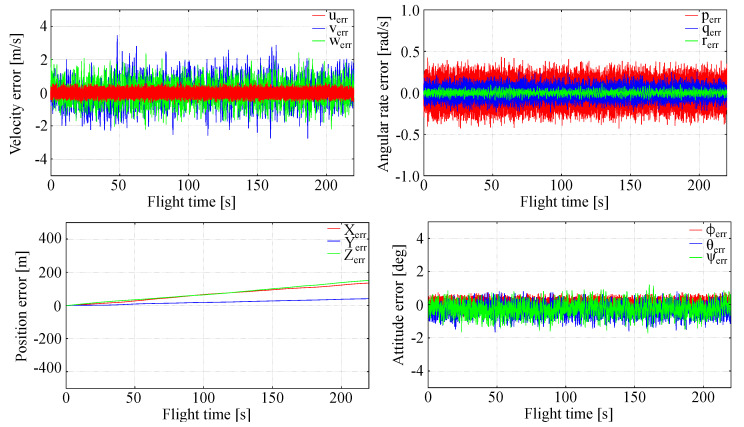
Full tracking errors—flight parameters disturbed by 2 units (Case 2).

In order to further analyze LQR control effectiveness, various disturbance levels were assumed and the results were compared to investigate the impact of disturbance on full tracking errors. The outcomes for a case when flight parameters were perturbed by 1 m/s (linear velocity), 1 rad/s (angular rates), 1 m (position) and 1${}^{\circ }$ (attitude) and a case when flight parameters were perturbed by 2 m/s (linear velocity), 2 rad/s (angular rates), 2 m (position) and 2${}^{\circ }$ (attitude) are presented in Figure 9 and Figure 10 and entitled Test 1 and Test 2, respectively. To assess the outcomes qualitatively, full tracking errors squared sum was evaluated for each flight parameter. Those results are presented in Table 4. The noise-free case presented in Figure 5 was included in the Table as well. As can be seen, increased disturbances lead to increased errors. The only way to decrease them is to retune *Q* and *R* weighting matrices.

In all test cases, the generalized LQR control applied to the full tracking problem appeared to be robust and stable. The tracking accuracy was very high, although it depended on the disturbance level and decreased smoothly with disturbances increasement. This behavior is acceptable from a practical point of view.

During the study, it turned out that the proper choice of weighting matrices was always possible. The gaps between lower and upper values of *Q* and *R* were of the order of 50% of their nominal values.

In the considered tracking problems, *Q* and *R* weighting matrices were fixed for the whole flight time. In all test cases, the same weighting matrices were used without the necessity of additional tuning.

## 6. Conclusions

In this paper, it has been shown that the LQR control can be used for a generalized tracking problem in aviation. It turned to be both accurate and robust, despite not taking into account physical disturbances explicitly. Those valuable properties of LQR are mainly due to its robustness. Even in case of inaccurate measurements or moderate physical disturbances, the LQR-based control had the ability for accurate full trajectory tracking. Moreover, it was able to compute the control positions of an aircraft with high accuracy.

This makes LQR control a valuable tool in the identification and tuning process of aircraft models based on real flight data for the purpose of high level full flight simulator certification.

In this study, a high accuracy flight dynamic model was used in order to limit the maximum unadded aircraft dynamics resulting from e.g., dynamic uncertainties or inputs degradation. A possible future research contribution is to modify the LQR control to take those features into account. Moreover, automatized weighting matrices for states and controls could be applied instead of the used approach.

## Figures and Tables

**Table 1:** Aircraft specification.

Feature	Value
max takeoff mass	120,000 kg
wingspan	42 m
wing area	220 m^2^
engines	4 turboprop, 2300 kW each
max speed	500 km/h
max range	6000 km
max altitude	8000 m

**Table 2:** Relative standard deviations, %.

$\hat{\Theta }$	OEM	NLS	$\hat{\Theta }$	OEM	NLS
$C_{L0}$	6.09	6.94	$C_{l_{0}}$	3.39	6.44
$C_{L_{WB_{\alpha }}}$	2.97	4.45	$C_{l_{\beta }}$	8.65	10.07
$C_{L_{H_{\alpha H}}}$	6.72	6.54	$C_{l_{p}}$	0.75	1.11
$C_{L_{H_{\delta H}}}$	2.80	3.48	$C_{l_{p\alpha }}$	9.04	10.06
$C_{D_{0}}$	7.67	1.02	$C_{l_{r}}$	1.10	4.03
$C_{m_{0}}$	1.90	5.99	$C_{l_{r\alpha }}$	4.42	18.62
$C_{m_{q}}$	1.65	8.05	$C_{l_{\delta A}}$	0.59	0.64
$C_{Y_{0}}$	8.13	14.41	$C_{l_{\delta R}}$	8.48	10.62
$C_{Y_{\beta }}$	3.12	0.17	$C_{n_{0}}$	7.69	2.62
$C_{Y_{p}}$	5.76	1.49	$C_{n_{\beta }}$	1.93	1.09
$C_{Y_{p\alpha }}$	4.84	5.95	$C_{n_{p}}$	1.08	10.89
$C_{Y_{r}}$	0.40	3.68	$C_{n_{p\alpha }}$	3.34	15.92
$C_{Y_{r\alpha }}$	9.45	3.79	$C_{n_{r}}$	0.35	0.72
$C_{Y_{\delta A}}$	0.56	0.17	$C_{n_{r\alpha }}$	5.53	8.33
$C_{Y_{\delta R}}$	0.73	0.61	$C_{n_{\delta R}}$	2.29	0.91

**Table 3:** Typical measurement errors in of nominal values percentage.

Variable	Unit	Error, %
Velocity (IAS)	m/s	2
Vertical velocity	m/s	5
Angle of attack and sideslip angle	∘	1
Angular rates	∘	1
Geographical coordinates	∘	5 × 10 ^−5^
Position (X,Y)	m	5
Altitude	m	5
Attitude angles	∘	1
Wind velocity	m/s	10
Wind direction	∘	5
Air temperature	${}^{\circ }$ C	5
Air pressure	Pa	5

**Table 4:** Squared full tracking errors sum.

	Noise-Free	Case 1	Case 2
$u_{err}$	0.0187	0.0867	0.1257
$v_{err}$	0.0139	0.3637	0.8103
$w_{err}$	0.0491	0.3242	0.6172
$p_{err}$	0.0041	0.1044	0.1589
$q_{err}$	0.0010	0.0529	0.0735
$r_{err}$	0.0002	0.0176	0.0284
$X_{err}$	1.5496	2.5515	4.1657
$Y_{err}$	0.6828	8.8825	14.6757
$Z_{err}$	0.6391	68.8531	123.8425
$\phi_{err}$	0.0237	0.1137	0.2237
$\theta_{err}$	0.0231	0.2822	0.5141
$\psi_{err}$	0.0060	0.1555	0.3600
